# P222: Period vs point prevalence – what is the difference?

**DOI:** 10.1186/2047-2994-2-S1-P222

**Published:** 2013-06-20

**Authors:** W Zingg, B Huttner, C Ginet, D Pittet

**Affiliations:** 1 Infection Control Programme, University of Geneva Hospitals, Geneva, Switzerland

## Introduction

Prevalence surveys serve as a rapid and simple means of obtaining healthcare associated infection (HAI) data. There is variability in following either the point or the period prevalence strategy, and whether the four major HAI categories (urinary tract infection [UTI], lower respiratory tract infection [LRI], bloodstream infection [BSI], surgical site infection [SSI]) should be reported rather than all HAI.

## Objectives

The aim of the study was to analyse the difference in outcome depending on the used survey strategy.

## Methods

The University of Geneva Hospital (HUG) is a primary and tertiary care centre at 8 hospital sites with 1908 beds, 48’112 admissions and 671’709 patient-days in 2011. Since 1994 yearly period prevalence surveys are performed. Surveillance includes all HAI as defined by the Centers for Disease Control and Prevention. The HUG surveillance allows to distinguish between period and point prevalence outcome from the same dataset. This study focused on the data between 2007 and 2012. Differences were analysed by stratifying the data by clinical setting (intensive care [ICU], acute care, subacute care, longterm care), and the group of HAI (UTI, LRI, BSI, SSI, other HAI [ENT-, skin-, gastrointestinal infections]).

## Results

The HAI prevalence for point and period methodology was 7.45% and 9.87% (+32%), respectively. The additional 33% (p<0.01) HAI were due to UTI (+52%), LRI (+31%), and other HAI (+40%). The difference for BSI (+15) and SSI (+19) was less important. HAI prevalence for point and period methodology in ICU, acute care, subacute care, and longterm care were 19.1%/24.1% (+25%), 6.3%/7.9% (+25%), 9.5%/13.4% (+41%), and 5.8%/8.9% (+53%), respectively. The differences were stable over time. Additional 43% LRI (p=0.05) and 79% (p=0.01) other HAI were detected by the period prevalence in longterm care, where the difference was most pronounced. The point and period prevalence the four major HAI categories were 5.88% and 7.65% (p<0.001), respectively.

## Conclusion

More HAI are identified by the period prevalence method. There is a tendency for period prevalence surveys to overestimate HAI of short duration (UTI; LRI; other HAI) and in subacute and longterm care settings. Point prevalence data cannot be benchmarked with period prevalence data.

## Disclosure of interest

None declared

